# Supplementary material for: Isoform-specific roles for AKT in affective behavior, spatial memory, and extinction related to psychiatric disorders
Source: eLife. 2020 Dec 16;9:e56630. doi: 10.7554/eLife.56630 (PMC7787664; doi:10.7554/eLife.56630)
Supplement: Supplementary file 1. [file elife-56630-supp1.docx]

**Supplementary file 1**

| **Figure** | ***Akt* Isoform** | **Homogeneity of variance test** | **Dependent Variable** | **Male** | **Female** | **N/group** |
| --- | --- | --- | --- | --- | --- | --- |
| **1A, B** | *Akt1* KO | Levene’s p=.086 M  Levene’s p=.308 F | Center time (%) | t(42)=4.789, p<.001 | t(27)=.598, p=.555 | WT-M=22  KO-M=22  WT-F=16  KO-F=13 |
|  |  | Levene’s p=.481 M  Levene’s p=.861 F | Distance moved (cm) | t(42)=.501 , p=.619 | t(27)=1.010, p=.321 |  |
|  | *Akt2* KO | Levene’s p=.302 M  Levene’s p=.216 F | Center time (%) | t(28)=-.841, p=.407 | t(22)=-.022, p=.983 | WT-M=14  KO-M=16  WT-F=13  KO-F=11 |
|  |  | Levene’s p=.037 M  Levene’s p=.965 F | Distance moved (cm) | t(26.087)=1.109, p=.277 | t(22)=.273, p=.788 |  |
|  | *Akt3* KO | Levene’s p=.285 M  Levene’s p=.319 F | Center time (%) | t(40)=-.964, p=.341 | t(34)=-.316, p=.754 | WT-M=19  KO-M=23  WT-F=18  KO-F=18 |
|  |  | Levene’s p=.387 M  Levene’s p=.707 F | Distance moved (cm) | t(40)=.219, p=.828 | t(34)=.864, p=.394 |  |
| **1C, D** | *Akt1* KO | Levene’s p=.207 M  Levene’s p=.617 F | Open Arm time (%) | t(42)=2.156, p=.037 | t(27)=-.302, p=.765 | WT-M=22  KO-M=22  WT-F=16  KO-F=13 |
|  |  | Levene’s p=.454 M  Levene’s p=.332 F | Distance moved (cm) | t(42)=.772, p=.445 | t(27)=-1.025, p=.314 |  |
|  | *Akt2* KO | Levene’s p=.140 M  Levene’s p=.441 F | Open Arm time (%) | t(26)=2.651, p=.013 | t(21)=-.550, p=.588 | WT-M=13  KO-M=15  WT-F=12  KO-F=11 |
|  |  | Levene’s p=.303 M  Levene’s p=.818 F | Distance moved (cm) | t(26)=-.659, p=.516 | t(21)=.194, p=.848 |  |
|  | *Akt3* KO | Levene’s p=.321 M  Levene’s p=.916 F | Open Arm time (%) | t(40)=.337, p=.738 | t(33)=-.103, p=.919 | WT-M=19  KO-M=23  WT-F=17  KO-F=18 |
|  |  | Levene’s p=.325 M  Levene’s p=.170 F | Distance moved (cm) | t(40)=.708, p=.483 | t(33)=-.691, p=.495 |  |

| **Figure** | ***Akt* Isoform** | **Homogeneity of variance test*** | **Dependent Variable** | **Male** | **Female** | **N/group** |
| --- | --- | --- | --- | --- | --- | --- |
| **2A, B** | *Akt1* KO  **Day1 values used for each phase* | Levene’s p=.091 M  Levene’s p=.233 F | Training Latency (s) | F(1,16)=.033, p=.859 | F(1,16)=.295, p=.595 | WT-M=9  KO-M=9  WT-F=9  KO-F=9 |
|  |  | Levene’s p=.649 M  Levene’s p=.553 F | Reversal Latency (s) | F(1,16)=.809, p=.382 | F(1,16)=1.265, p=.277 |  |
|  |  | Levene’s p=.067 M  Levene’s p=.180 F | Visible Latency (s) | F(1,16)=2.524, p=.132 | F(1,16)=1.423, p=.250 |  |
|  |  | Levene’s p=.447 M  Levene’s p=.055 F | Probe Quad Time (%) | t(16)=1.187, p=.253 | t(16)=-.489, p=.631 |  |
|  |  | Levene’s p=.118 M Levene’s p=.254 F | Platform Crossings (#) | t(16)=2.389, p=.030 | t(16)=-.160, p=.875 |  |
|  |  | Levene’s p=.261 M  Levene’s p=.979 F | Distance Swam (cm) | t(16)=-.307, p=.763 | t(16)=-.669, p=.513 |  |
|  |  | Levene’s p=.245 M  Levene’s p=.823 F | Velocity (cm/s) | t(16)=.005, p=.785 | t(16)=-.715, p=.485 |  |
| **2C, D** | *Akt2* KO  **Day1 values used for each phase* | Levene’s p=.950 M  Levene’s p=.112 F | Training Latency (s) | F(1,18)=.073, p=.790 | F(1,18)=4.267, p=.054 | WT-M=9  KO-M=11  WT-F=11  KO-F=9 |
|  |  | Levene’s p=.806 M  Levene’s p=.175 F | Reversal Latency (s) | F(1,18)=1.516, p=.234 | F(1,18)=.110, p=.743 |  |
|  |  | Levene’s p=.490 M  Levene’s p=.056 F | Visible Latency (s) | F(1,18)=.271, p=.609 | F(1,18)=.057, p=.814 |  |
|  |  | Levene’s p=.953 M  Levene’s p=.060 F | Probe Quad Time (%) | t(18)=.347, p=.733 | t(18)=-.004, p=.997 |  |
|  |  | Levene’s p=.433 M  Levene’s p=.006 F | Platform Crossings (#) | t(18)=.124, p=.903 | t(13.557)=-.327, p=.748 |  |
|  |  | Levene’s p=.755 M  Levene’s p=.382 F | Distance Swam (cm) | t(18)=-.127, p=.900 | t(18)=-1.037, p=.314 |  |
|  |  | Levene’s p=.794 M  Levene’s p=.220 F | Velocity (cm/s) | t(18)=-.086, p=.933 | t(18)=-.219, p=.829 |  |
| **2E, F** | *Akt3* KO  **Day1 values used for each phase* | Levene’s p=.577 M  Levene’s p=.346 F | Training Latency (s) | F(1,19)=.010, p=.923 | F(1,17)=.429, p=.521 | WT-M=10  KO-M=11  WT-F=10  KO-F=9 |
|  |  | Levene’s p=.589 M  Levene’s p=.367 F | Reversal Latency (s) | F(1,19)=.196, p=.663 | F(1,17)=2.306, p=.147 |  |
|  |  | Levene’s p=.189 M  Levene’s p=.129 F | Visible Latency (s) | F(1,19)=1.785, p=.197 | F(1,17)=1.090, p=.311 |  |
|  |  | Levene’s p=.844 M  Levene’s p=.614 F | Probe Quad Time (%) | t(19)=.838, p=.412 | t(17)=.113, p=.912 |  |
|  |  | Levene’s p=.158 M  Levene’s p=.364 F | Platform Crossings (#) | t(19)=.246, p=.808 | t(17)=.227, p=.823 |  |
|  |  | Levene’s p=.500 M  Levene’s p=.448 F | Distance Swam (cm) | t(19)=-1.231, p=.233 | t(17)=1.201, p=.246 |  |
|  |  | Levene’s p=.498 M  Levene’s p=.447 F | Velocity (cm/s) | t(19)=-1.231, p=.234 | t(17)=1.200, p=.246 |  |

| **Figure** | ***Akt* Isoform** | **Homogeneity of variance test** | **Dependent Variable** | **Males** | **Females** | **N/group** |
| --- | --- | --- | --- | --- | --- | --- |
| **3A, B** | *Akt1* KO | Levene’s p=.004 M  Levene’s p=.112 F | Training pre-CS freezing (%) | t(16.157)=-2.143, p=.047 | t(18)=-.713, p=.485 | WT-M=16-17  KO-M=13-15  WT-F=10  KO-F=10 |
|  |  | Levene’s p=.129 M  Levene’s p=.434 F | Training freezing (%) | F(1,27)=2.193, p=.150 | F(1,18)=.567, p=.461 |  |
|  |  | Levene’s p=.030 M  Levene’s p=.139 F | Context LTM freezing (%) | t(26.588)=-1.699, p=.101 | t(18)=1.216, p=.240 |  |
|  |  | Levene’s p=.019 M  Levene’s p=.078 F | Cued LTM pre-CS freezing (%) | t(16.323)=-2.558, p=.021 | t(18)=1.616, p=.124 |  |
|  |  | Levene’s p=.564 M  Levene’s p=.587 F | Cued LTM freezing (%) | F(1,30)=.000, p=.984 | F(1,18)=.463, p=.505 |  |
| **3C, D** | *Akt2* KO | Levene’s p=.723 M  Levene’s p=.136 F | Training pre-CS freezing (%) | t(26)=.163, p=.872 | t(18)=-.650, p=.524 | WT-M=14  KO-M=14-16  WT-F=10-11  KO-F=10 |
|  |  | Levene’s p=.024 M  Levene’s p=.077 F | Training freezing (s) | F(1,17.90)=2.354, p=.137 | F(1,18)=.376, p=.547 |  |
|  |  | Levene’s p=.488 M  Levene’s p=.108 F | Context LTM freezing (%) | t(28)=-2.474, p=.020 | t(19)=1.436, p=.167 |  |
|  |  | Levene’s p=.236 M  Levene’s p=.331 F | Cued LTM pre-CS freezing (%) | t(28)=.520, p=.607 | t(19)=.742, p=.467 |  |
|  |  | Levene’s p=.107 M  Levene’s p=.331 F | Cued LTM freezing (%) | F(1,28)=.197, p=.660 | F(1,19)=.000, p=.997 |  |
| **3E, F** | *Akt3* KO | Levene’s p=.000 M  Levene’s p=.002 F | Training pre-CS freezing (%) | t(17.547)=-3.012, p=.008 | t(17.631)=2.201, p=.041 | WT-M=18  KO-M=18-20  WT-F=17  KO-F=16-17 |
|  |  | Levene’s p=.013 M  Levene’s p=.329 F | Training freezing (%) | F(1,25.299)=2.738, p=.107 | F(1,31)=.004, p=.951 |  |
|  |  | Levene’s p=.762 M  Levene’s p=.186 F | Context LTM freezing (%) | t(36)=-1.339, p=.189 | t(32)=-.660, p=.514 |  |
|  |  | Levene’s p=.006 M  Levene’s p<.001 F | Cued LTM pre-CS freezing (%) | t(22.00)=-1.569, p=.131 | t(16.698)=-1.890, p=.076 |  |
|  |  | Levene’s p=.143 M  Levene’s p=.598 F | Cued LTM freezing (%) | F(1,36)=.253, p=.618 | F(1,32)=.177, p=.677 |  |

| **Figure** | ***Akt* Isoform** | **Homogeneity of variance test*** | **Dependent Variable** | **Males** | **Females** | **N/group** |
| --- | --- | --- | --- | --- | --- | --- |
| **4A, B** | *Akt1* KO  **Day1 values used* | Levene’s p=.264 M  Levene’s p=.036 F | D1 pre-CS freezing (%) | t(30)=1.028, p=.312 | t(15.165)=1.394, p=.183 | WT-M=17  KO-M=15  WT-F=10  KO-F=10 |
|  |  | Levene’s p=.181 M  Levene’s p=.095 F | D1-D3 freezing (%) | F(1,30)=8.571, p=.006 | F(1,18)=.509, p=.485 |  |
|  |  | Levene’s p=.264 M  Levene’s p=.029 F | Ext LTM pre-CS freezing (%) | t(30)=1.496, p=.145 | t(12.463)=-1.754, p=.104 |  |
|  |  | Levene’s p=.609 M  Levene’s p=.064 F | Ext LTM freezing (%) | F(1,30)=9.121, p=.005 | F(1,18)=.247, p=.625 |  |
|  |  | Levene’s p=.001 M  Levene’s p=.127 F | Renewal pre-CS freezing (%) | t(17.452)=3.010, p=.008 | t(18)=.451, p=.657 |  |
|  |  | Levene’s p=.228 M  Levene’s p=.318 F | Renewal freezing (%) | F(1,30)=7.187, p=.012 | F(1,18)=1.337, p=.263 |  |
| **4C, D** | *Akt2* KO  **Day1 values used* | Levene’s p=.007 M  Levene’s p=.980 F | D1 pre-CS freezing (%) | t(16.911)=1.262, p=.224 | t(16)=-.423, p=.678 | WT-M=13-14  KO-M=15-16  WT-F=9  KO-F=9 |
|  |  | Levene’s p=.266 M  Levene’s p=.214 F | D1-D3 freezing (%) | F(1,28)=1.795, p=.191 | F(1,14)=.098, p=.759 |  |
|  |  | Levene’s p=.032 M  Levene’s p=.071 F | Ext LTM pre-CS freezing (%) | t(18.150)=1.421, p=.172 | t(16)=-.943, p=.360 |  |
|  |  | Levene’s p=.074 M  Levene’s p=.895 F | Ext LTM freezing (%) | F(1,28)=.102, p=.752 | F(1,16)=.045, p=.835 |  |
|  |  | Levene’s p=.474 M  Levene’s p=.400 F | Renewal pre-CS freezing (%) | t(26)=.527, p=602 | t(16)=-.709, p=.489 |  |
|  |  | Levene’s p=.432 M  Levene’s p=.405 F | Renewal freezing (%) | F(1,26)=5.969, p=.022 | F(1,16)=.231, p=.637 |  |
| **4E, F** | *Akt3* KO  **Day1 values used* | Levene’s p=.726 M  Levene’s p=.001 F | D1 pre-CS freezing (%) | t(36)=-.798, p=.430 | t(19.972)=-1.957, p=.065 | WT-M=18  KO-M=20  WT-F=15-17  KO-F=15-16 |
|  |  | Levene’s p=.205 M  Levene’s p=.567 F | D1-D3 freezing (%) | F(1,34)=1.032, p=.317 | F(1,28)=.816, p=.374 |  |
|  |  | Levene’s p=.571 M  Levene’s p=.099 F | Ext LTM pre-CS freezing (%) | t(36)=.241, p=.811 | t(29)=-1.049, p=.303 |  |
|  |  | Levene’s p=.429 M  Levene’s p=.221 F | Ext LTM freezing (%) | F(1,36)=.205, p=.653 | F(1,29)=.412, p=.526 |  |
|  |  | Levene’s p=.358 M  Levene’s p=.007 F | Renewal pre-CS freezing (%) | t(36)=-1.504 p=.141 | t(22.686)=-1.089, p=.288 |  |
|  |  | Levene’s p=.127 M  Levene’s p=.091 F | Renewal freezing (%) | F(1,36)=.031, p=.861 | F(1,30)=.239, p=.629 |  |

| **Figure** | ***Akt* Isoform** | **Homogeneity of variance test** | **Dependent Variable** | **Genotype (males only)** | **Post hoc testing (Tukey)** | **N/group** |
| --- | --- | --- | --- | --- | --- | --- |
| **5D** | *Akt1* KO +  *AAV-AKT1* | Levene’s p=.389 | Training pre-CS freezing (%) | F(2,34)=.209, p=.812 | All comparisons p>.05 | WT-Sham=15  KO-Sham=11  KO-*AKT1*=11 |
|  |  | Levene’s p=.571 | Training freezing (%) | F(2,34)=.058, p=.944 | All comparisons p>.05 |  |
|  |  | Levene’s p=.421 | Context LTM freezing (%) | F(2,34)=.023, p=.977 | All comparisons p>.05 |  |
|  |  | Levene’s p=.096 | Cued LTM pre-CS freezing (%) | F(2,34)=2.033, p=.146 | All comparisons p>.05 |  |
|  |  | Levene’s p=.996 | Cued LTM freezing (%) | F(2,34)=1.150, p=.329 | All comparisons p>.05 |  |
| **5E** | *Akt1* KO *+*  *AAV-AKT1* | Levene’s p=.299 | D1 pre-CS freezing (%) | F(2,34)=.463, p=.633 | All comparisons p>.05 | WT-Sham=15  KO-Sham=11  KO-*AKT1*=11 |
|  |  | Levene’s p=.119 | D1-D3 freezing (%) | F(2,34)=5.400, p=.009 | WT vs KO p=.009  WT vs KO-*AKT1* p=.875  KO vs KO-*AKT1* p=.047 |  |
|  |  | Levene’s p=.165 | Ext LTM pre-CS freezing (%) | F(2,34)=.994, p=.381 | All comparisons p>.05 |  |
|  |  | Levene’s p=.096 | Ext LTM freezing (%) | F(2,34)=4.388, p=.020 | WT vs KO p=.035  WT vs KO-*AKT1* p=.985  KO vs KO-*AKT1* p=.037 |  |
|  |  | Levene’s p=.790 | Renewal pre-CS freezing (%) | F(2,29)=.117, p=.890 | All comparisons p>.05 | WT-Sham=13  KO-Sham=11  KO-*AKT1*=8 |
|  |  | Levene’s p=.243 | Renewal freezing (%) | F(2,29)=1.018, p=.374 | All comparisons p>.05 |  |

| **Figure** | ***Akt* Isoform** | **Homogeneity of variance test*** | **Dependent Variable** | **Genotype (males only)** | **N/group** |
| --- | --- | --- | --- | --- | --- |
| **6A** | *Akt1* cKO | Levene’s p=.205 | Center time (%) | t(32)=.157, p=.876 | WT=14  cKO=20 |
|  |  | Levene’s p=.074 | Distance moved (cm) | t(32)=-.681, p=.501 |  |
| **6B** | *Akt1* cKO | Levene’s p=.565 | Open Arm time (%) | t(32)=-.203, p=.840 |  |
|  |  | Levene’s p=.062 | Distance moved (cm) | t(32)=.441, p=.662 |  |
| **6C** | *Akt1* cKO  **Day1 values used for each phase* | Levene’s p=.167 | Training Latency (s) | F(1,14)=.111, p=.744 | WT=8  cKO=8 |
|  |  | Levene’s p=.664 | Reversal Latency (s) | F(1,14)=.160, p=.695 |  |
|  |  | Levene’s p=.514 | Visible Latency (s) | F(1,14)=1.00, p=.334 |  |
|  |  | Levene’s p=.499 | Probe Quad Time (s) | t(14)=.92, p=.928 |  |
|  |  | Levene’s p=1.00 | Platform Crossings (#) | t(14)=.914 p=.376 |  |
|  |  | Levene’s p=.848 | Distance Swam (cm) | t(14)=.250 p=.806 |  |
|  |  | Levene’s p=.846 | Velocity (cm/s) | t(14)=.414 p=.685 |  |
| **6D** | *Akt1* cKO | Levene’s p=.066 | Training pre-CS freezing (%) | t(22)=.854, p=.402 | WT=11  cKO=13 |
|  |  | Levene’s p=.187 | Training freezing (%) | F(1,22)=.035, p=.853 |  |
|  |  | Levene’s p=.918 | Context LTM freezing (%) | t(22)=2.535, p=.019 |  |
|  |  | Levene’s p=.315 | Cued LTM pre-CS freezing (%) | t(22)=1.331, p=.197 |  |
|  |  | Levene’s p=.098 | Cued LTM freezing (%) | F(1,22)=.677, p=.419 |  |
| **6E** | *Akt1* cKO | Levene’s p=.220 | D1 pre-CS freezing (%) | t(22)=1.106, p=.281 | WT=11  cKO=13 |
|  |  | Levene’s p=.318 | D1-D3 freezing (%) | F(1,22)=1.009, p=.326 |  |
|  |  | Levene’s p=.808 | Ext LTM pre-CS freezing (%) | t(22)=.168, p=.868 |  |
|  |  | Levene’s p=.675 | Ext LTM freezing (%) | F(1,22)=.917, p=.349 |  |
|  |  | Levene’s p=.130 | Renewal pre-CS freezing (%) | t(22)=1.548, p=.136 |  |
|  |  | Levene’s p=.360 | Renewal freezing (%) | F(1,22)=3.105, p=.092 |  |
| **supplement 1** | *Akt1* cKO | Levene’s p=.467 | Amygdala AKT1 levels | t(10)=6.039, p<.001 | WT=6  cKO=6 |
|  |  | Levene’s p=.765 | Amygdala AKT3 levels | t(10)=.395, p=.701 |  |
|  |  | Levene’s p=.006 | PFC AKT1 levels | t(10)=6.705, p<.001 |  |
|  |  | Levene’s p=.651 | PFC AKT3 levels | t(10)=.503, p=.626 |  |

| **Figure** | ***Akt* Isoform** | **Homogeneity of variance test*** | **Dependent Variable** | **Males** | **Females** | **N/group** |
| --- | --- | --- | --- | --- | --- | --- |
| **7A** | *Akt1* cKO *Akt3* KO | Levene’s p=.402 M  Levene’s p=.123 F | Center time (s) | t(26)=.314, p=.757 | t(29)=1.404, p=.171 | WT-M=15  cKO-M=13  WT-F=16  cKO-F=15 |
|  |  | Levene’s p=.088 M  Levene’s p=.158 F | Distance moved (cm) | t(26)=.106, p=.917 | t(29)=-1.849, p=.075 |  |
| **7B** | *Akt1* cKO *Akt3* KO | Levene’s p=.599 M  Levene’s p=.973 F | Open Arm time (%) | t(26)=-.498, p=.622 | t(29)=-.613, p=.545 |  |
|  |  | Levene’s p=.390 M  Levene’s p=.328 F | Distance moved (cm) | t(26)=-.385, p=.703 | t(29)=-1.821, p=.079 |  |
| **7C, D** | *Akt1* cKO *Akt3* KO  **Day1 values used for each phase* | Levene’s p=.345 M  Levene’s p=.506 F | Training Latency (s) | F(1,20)=.220, p=.644 | F(1,22)=2.159, p=.156 | WT-M=13  cKO-M=9  WT-F=12  cKO-F=12 |
|  |  | Levene’s p=.299 M  Levene’s p=.112 F | Reversal Latency (s) | F(1,20)=.070, p=.794 | F(1,22)=1.936, p=.178 |  |
|  |  | Levene’s p=.783 M  Levene’s p=.197 F | Visible Latency (s) | F(1,20)=.012, p=.916 | F(1,22)=.741, p=.399 |  |
|  |  | Levene’s p=.861 M  Levene’s p=.336 F | Probe Quad Time (s) | t(20)=2.172, p=.042 | t(22)=3.262, p=.004 |  |
|  |  | Levene’s p=.176 M  Levene’s p=.284 F | Platform Crossings (#) | t(20)=3.406, p=.003 | t(22)=2.283, p=.032 |  |
|  |  | Levene’s p=.453 M  Levene’s p=.617 F | Distance Swam (cm) | t(20)=.568, p=.576 | t(22)=-.642, p=.528 |  |
|  |  | Levene’s p=.640 M  Levene’s p=.565 F | Velocity (cm/s) | t(20)=.772, p=.449 | t(22)=-.359, p=.723 |  |
| **7E, F** | *Akt1* cKO *Akt3* KO | Levene’s p=.003 M  Levene’s p=.092 F | Training pre-CS freezing (%) | t(10.594)=1.566, p=.147 | t(23)=-1.250, p=.224 | WT-M=11  cKO-M=11  WT-F=14  cKO-F=10-11 |
|  |  | Levene’s p=.844 M  Levene’s p=.489 F | Training freezing (%) | F(1,20)=.163, p=.690 | F(1,23)=.011, p=.919 |  |
|  |  | Levene’s p=.400 M  Levene’s p=.958 F | Context LTM freezing (%) | t(20)=2.213, p=.039 | t(23)=-2.250, p=.034 |  |
|  |  | Levene’s p=.612 M  Levene’s p=.474 F | Cued LTM pre-CS freezing (%) | t(20)=-.672, p=.509 | t(22)=.468, p=.644 |  |
|  |  | Levene’s p=.864 M  Levene’s p=.930 F | Cued LTM freezing (%) | F(1,20)=5.176, p=.034 | F(1,22)=4.533, p=.045 |  |

| **Figure** | ***Akt* Isoform** | **Homogeneity of variance test** | **Dependent Variable** | **Male vs Female (WT only)** | **Male only** | **Female only** | **N/group** |
| --- | --- | --- | --- | --- | --- | --- | --- |
| **8A** | WT | Levene’s p=.909 | AKT1 | t(19)=.418, p=.681 |  |  | AKT1, AKT2, pan AKT  WT-M=10  WT-F=11  AKT3  WT-M=9  WT-F=10 |
|  |  | Levene’s p=.968 | AKT2 | t(19)=-3.045, p=.007 |  |  |  |
|  |  | Levene’s p=.430 | AKT3 | t(17)=.913, p=.374 |  |  |  |
|  |  | Levene’s p=.898 | pan AKT | t(19)=.189, p=.852 |  |  |  |
|  |  | Levene’s p=.737 | pAKT1 | t(19)=-.030, p=.976 |  |  |  |
|  |  | Levene’s p=.855 | pAKT2 | t(19)=.339, p=.738 |  |  |  |
|  |  | Levene’s p=.973 | pan pAKT | t(19)=-.331, p=.745 |  |  |  |
| **8B, C** | *Akt1* KO | Levene’s p=.456 M  Levene’s p=.544 F | pan AKT |  | t(12)=3.427, p=.005 | t(14)=3.507, p=.004 | WT-M=7  KO-M=7  WT-F=8  KO-F=8 |
|  |  | Levene’s p=.415 M  Levene’s p=.984 F | pan pAKT |  | t(12)=-2.541, p=.026 | t(14)=- 3.456, p=.004 |  |
|  | *Akt2* KO | Levene’s p=.154 M  Levene’s p=.258 F | pan AKT |  | t(12)=2.282, p=.042 | t(10)=2.253, p=.048 | WT-M=7  KO-M=7  WT-F=6  KO-F=6 |
|  |  | Levene’s p=.888 M  Levene’s p=.559 F | pan pAKT |  | t(12)=1.222, p=.245 | t(10)=.403, p=.695 |  |
|  | *Akt3* KO | Levene’s p=.750 M  Levene’s p=.655 F | pan AKT |  | t(8)=11.793, p<.001 | t(7)=11.773, p<.001 | WT-M=5  KO-M=5  WT-F=4  KO-F=5 |
|  |  | Levene’s p=.172.M  Levene’s p=.145.F | pan pAKT |  | t(8)=-.222, p=.830 | t(7)=.259,  p=.803 |  |
| **8D** | *Akt1* KO | Levene’s p=.725 M  Levene’s p=.697 F | pAKT2 |  | t(12)=-2.645, p=.021 | t(14)=-2.160, p=.049 | WT-M=7  KO-M=7  WT-F=8  KO-F=8 |
| **8E** | *Akt3* KO | Levene’s p=.280 M  Levene’s p=.007 F | pAKT1 |  | t(8)=-3.130, p=.014 | t(15)=-3.724, p=.002 | WT-M=5  KO-M=5  WT-F=8  KO-F=9 |

| **Figure** | ***Akt* Isoform** | **Brain Region** | **Homogeneity of variance test** | **Dependent Variable** | **Male** | **Female** | **N/group** |
| --- | --- | --- | --- | --- | --- | --- | --- |
| **9A, B** | *Akt1* KO | Hippocampus | Levene’s p=.094 M  Levene’s p=.205 F | pPDK1 | t(12)=-.336, p=.742 | t(12)=-.537, p=.601 | WT-M=7  KO-M=7  WT-F=7  KO-F=7 |
|  |  |  | Levene’s p=.730 M  Levene’s p=.074 F | PDK1 | t(12)=.103, p=.920 | t(12)=-.859, p=.407 |  |
|  |  |  | Levene’s p=.064 M  Levene’s p=.909 F | pGSK3β | t(12)=-.863, p=.405 | t(12)=.382, p=.709 |  |
|  |  |  | Levene’s p=.142 M  Levene’s p=.531 F | GSK3β | t(12)=-.340, p=.740 | t(12)=-1.079, p=.302 |  |
|  |  |  | Levene’s p=.563 M  Levene’s p=.772 F | pERK1/2 | t(12)=.414, p=.686 | t(12)=-.587, p=.568 |  |
|  |  |  | Levene’s p=.461 M  Levene’s p=.068 M | ERK1/2 | t(12)=.041, p=.968 | t(12)=-.344, p=.737 |  |
| **9C, D** | *Akt1* KO | PFC | Levene’s p=.658 M  Levene’s p=.615 F | pPDK1 | t(10)=.596, p=.564 | t(10)=1.093, p=.564 | pPDK1, pERK1/2  WT-M=6  KO-M=6  WT-F=6  KO-F=6  pGSK3β  WT-M=8  KO-M=8  WT-F=7  KO-F=7 |
|  |  |  | Levene’s p=.968 M  Levene’s p=.687 F | PDK1 | t(10)=-.195, p=.850 | t(10)=.073, p=.943 |  |
|  |  |  | Levene’s p=.127 M  Levene’s p=.881 F | pGSK3β | t(12)=-.433, p=.672 | t(12)=3.567, p=.004 |  |
|  |  |  | Levene’s p=.479 M  Levene’s p=.410 F | GSK3β | t(12)=-.812 , p=.430 | t(12)=-.330, p=.747 |  |
|  |  |  | Levene’s p=.784 M  Levene’s p=.477 F | pERK1/2 | t(10)=.039, p=.969 | t(10)=.119, p=.907 |  |
|  |  |  | Levene’s p=.965 M  Levene’s p=.064 F | ERK1/2 | t(10)=-1.005, p=.339 | t(10)=.521, p=.614 |  |
| **9E, F** | *Akt2* KO | Hippocampus | Levene’s p=.367 M  Levene’s p=.195 F | pPDK1 | t(8)=.804, p=.445 | t(8)=.630, p=.546 | WT-M=5  KO-M=5  WT-F=5  KO-F=5 |
|  |  |  | Levene’s p=.999 M  Levene’s p=.084 F | PDK1 | t(8)=-.451, p=.664 | t(8)=-.517, p=.619 |  |
|  |  |  | Levene’s p=.062 M  Levene’s p=.430 F | pGSK3β | t(8)=.605, p=.562 | t(8)=-.573, p=.582 |  |
|  |  |  | Levene’s p=.324 M  Levene’s p.081.F | GSK3β | t(8)=1.041, p=.328 | t(8)=-.063, p=.951 |  |
|  |  |  | Levene’s p=.141 M  Levene’s p=.927 F | pERK1/2 | t(8)=.943, p=.373 | t(8)=-.696, p=.506 |  |
|  |  |  | Levene’s p=.544 M  Levene’s p=.174 F | ERK1/2 | t(8)=.601, p=.565 | t(8)=.377, p=.716 |  |
| **supplement 2** | *Akt3* KO | Hippocampus | Levene’s p=.149 | pGSK3β |  | t(8)=.296, p=.775 | WT-F=5  KO-F=5 |
|  |  |  | Levene’s p=.172 | GSK3β |  | t(8)=-1.120, p=.295 |  |
| **supplement 3B** | WT | PFC | Levene’s p=.685 | PV+ cells | Male vs Female (WT only)  t(4)=-.335, p=.754 | | WT-M=3  WT-F=3 |
| **supplement 3C** | WT | PFC | Levene’s p=.06 | GAD67 | Male vs Female (WT only) t(10)=1.480, p=.170 | | WT-M=6  WT-F=6 |
