# Supplementary material for: Isoform-specific roles for AKT in affective behavior, spatial memory, and extinction related to psychiatric disorders
Source: eLife. 2020 Dec 16;9:e56630. doi: 10.7554/eLife.56630 (PMC7787664; doi:10.7554/eLife.56630)
Supplement: Supplementary file 2. [file elife-56630-supp2.docx]

**Supplementary file 2**

| **Figure** | **Outliers** | **Exclusion Criterion** |
| --- | --- | --- |
| **1C,1D** | 1 *Akt2* WT male, 1 *Akt2* KO male,  1 *Akt2* WT female, 1 *Akt3* WT female | Grubbs’ method (distance moved) |
| **3A,3B** | 3 WT males, 1 KO male | Equipment error |
| **3C,3D** | 2 KO males, 1 WT female | Equipment error |
| **3E,3F** | 1 WT male, 2 KO males, 1 KO female | Equipment error |
| **4A,4B** | 3 WT males, 1 KO male | Equipment error |
| **4C,4D** | 1 WT male, 1 KO male,  2 WT females, 1 KO female | Equipment error |
| **4E,4F** | 1 WT male,  2 WT females, 2 KO females | Equipment error |
| **5D** | 1 *Akt1* KO-sham | Surgical exclusion based on GFP expression |
| **5E** | 2 *Akt1* WT-sham,  3 *Akt1* KO-*AKT1* | Subjects died pre-renewal testing |
| **6D,6E** | 1 *Akt1* cKO | Grubbs’ method (pre-CS freezing) |
| **7A,7B** | 1 *Akt1* cKO *Akt3* KO female | Grubbs’ method (distance moved) |
| **7C** | 1 *Akt1* cKO *Akt3* KO male | Failed to meet training criterion of 50% of D1 latency by D8 |
| **7F** | 1 *Akt1* cKO *Akt3* KO female | Equipment error |
| **8** | 1 *Akt3* WT female | Grubbs’ method (signal intensity) |
| **9-figure supplement 2** | 1 *Akt3* WT female  1 *Akt3* KO female | Grubbs’ method (signal intensity) |
