# Supplementary material for: Isoform-specific roles for AKT in affective behavior, spatial memory, and extinction related to psychiatric disorders
Source: eLife. 2020 Dec 16;9:e56630. doi: 10.7554/eLife.56630 (PMC7787664; doi:10.7554/eLife.56630)
Supplement: Supplementary file 3. [file elife-56630-supp3.docx]

**Supplementary file 3**

| **Figure** | ***Akt* Isoform** | **Homogeneity of variance test** | **Dependent Variable** | **Genotype** | **Sex** | **Interaction (Sex x Genotype)** | **N/group** |
| --- | --- | --- | --- | --- | --- | --- | --- |
| **1A, B** | *Akt1* KO | Levene’s p=.225 | Center time (%) | F(1,69)=10.457 p=.002 | F(1,69)=3.542  p=.064 | F(1,69)=4.721  p=.033 | WT-M=22  KO-M=22  WT-F=16  KO-F=13 |
|  |  | Levene’s p=.170 | Distance moved (cm) | F(1,69)=1.037  p=.312 | F(1,69)=2.544 p=.115 | F(1,69)=0.116  p=.734 |  |
|  | *Akt2* KO | Levene’s p=.144 | Center time (%) | F(1,50)=.266 p=.608 | F(1,50)=1.116  p=.296 | F(1,50)=.230  p=.634 | WT-M=14  KO-M=16  WT-F=13  KO-F=11 |
|  |  | Levene’s p=.339 | Distance moved (cm) | F(1,50)=.777  p=.382 | F(1,50)=2.095 p=.154 | F(1,50)=.188  p=.666 |  |
|  | *Akt3* KO | Levene’s p=.143 | Center time (%) | F(1,74)=.656  p=.421 | F(1,74)=5.671 p=.020 | F(1,74)=0.070  p=.792 | WT-M=19  KO-M=23  WT-F=18  KO-F=18 |
|  |  | Levene’s p=.839 | Distance moved (cm) | F(1,74)=.661  p=.419 | F(1,74)=.933  p=.337 | F(1,74)=.283  p=.597 |  |
| **1C, D** | *Akt1* KO | Levene’s p=.361 | Open Arm time (%) | F(1,69)=1.608  p=.209 | F(1,69)=2.298 p=.134 | F(1,69)=2.785  p=.100 | WT-M=22  KO-M=22  WT-F=16  KO-F=13 |
|  |  | Levene’s p=.083 | Distance moved (cm) | F(1,69)=.002  p=.967 | F(1,69)=1.322 p=.254 | F(1,69)=1.305  p=.257 |  |
|  | *Akt2* KO | Levene’s p=.271 | Open Arm time (%) | F(1,47)=2.287 p=.137 | F(1,47)=.005  p=.947 | F(1,47)=5.118  p=.028 | WT-M=13  KO-M=15  WT-F=12  KO-F=11 |
|  |  | Levene’s p=.131 | Distance moved (cm) | F(1,47)=.014  p=.906 | F(1,47)=.732 p=.397 | F(1,47)=.240  p=.627 |  |
|  | *Akt3* KO | Levene’s p=.719 | Open Arm time (%) | F(1,73)=.023  p=.881 | F(1,73)=.467  p=.497 | F(1,73)=.092  p=.763 | WT-M=19  KO-M=23  WT-F=17  KO-F=18 |
|  |  | Levene’s p=.300 | Distance moved (cm) | F(1,73)=.000  p=.984 | F(1,73)=.586  p=.446 | F(1,73)=.973  p=.327 |  |

| **Figure** | ***Akt* Isoform** | **Homogeneity of variance test*** | **Dependent Variable** | **Genotype** | **Sex** | **Interaction (Sex x Genotype)** | **N/group** |
| --- | --- | --- | --- | --- | --- | --- | --- |
| **2A, B** | *Akt1* KO  **Day1 values used for each phase* | Levene’s p=.211 | Training Latency (s) | F(1,32)=.076 p=.784 | F(1,32)=8.198  p=.007 | F(1,32)=.272  p=.606 | WT-M=9  KO-M=9  WT-F=9  KO-F=9 |
|  |  | Levene’s p=.134 | Reversal Latency (s) | F(1,32)=2.074  p=.160 | F(1,32)=.984  p=.329 | F(1,32)=.117  p=.735 |  |
|  |  | Levene’s p=.170 | Visible Latency (s) | F(1,32)=.174  p=.679 | F(1,32)=.351  p=.557 | F(1,32)=3.061  p=.090 |  |
|  |  | Levene’s p=.767 | Probe Quad Time (%) | F(1,32)=.127  p=.724 | F(1,32)=.287 p=.586 | F(1,32)=4.368  p=.045 |  |
|  |  | Levene’s p=.073 | Platform Crossings (#) | F(1,32)=4.577 p=.040 | F(1,32)=3.783  p=.061 | F(1,32)=9.683  p=.004 |  |
|  |  | Levene’s p=.055 | Distance Swam (cm) | F(1,32)=.003  p=.954 | F(1,32)=8.245 p=.007 | F(1,32)=.008  p=.928 |  |
|  |  | Levene’s p=.258 | Velocity (cm/s) | F(1,32)=.024  p=.877 | F(1,32)=9.135 p=.005 | F(1,32)=.026  p=.874 |  |
| **2C, D** | *Akt2* KO  **Day1 values used for each phase* | Levene’s p=.360 | Training Latency (s) | F(1,36)=.359  p=.553 | F(1,36)=3.392  p=.074 | F(1,36)=1.193  p=.282 | WT-M=9  KO-M=11  WT-F=11  KO-F=9 |
|  |  | Levene’s p=.114 | Reversal Latency (s) | F(1,36)=.897  p=.350 | F(1,36)=5.223  p=.028 | F(1,36)=.151  p=.700 |  |
|  |  | Levene’s p=.141 | Visible Latency (s) | F(1,36)=.008  p=.930 | F(1,36)=.929  p=.342 | F(1,36)=.236  p=.630 |  |
|  |  | Levene’s p=.057 | Probe Quad Time (%) | F(1,36)=.069 p=.794 | F(1,36)=.022 p=.884 | F(1,36)=.066 p=.799 |  |
|  |  | Levene’s p=.075 | Platform Crossings (#) | F(1,36)=.084 p=.773 | F(1,36)=.001 p=.977 | F(1,36)=.010 p=.920 |  |
|  |  | Levene’s p=.052 | Distance Swam (cm) | F(1,36)=.151 p=.700 | F(1,36)=4.326 p=.045 | F(1,36)=.021 p=.887 |  |
|  |  | Levene’s p=.111 | Velocity (cm/s) | F(1,36)=.018 p=.895 | F(1,36)=5.621 p=.023 | F(1,36)=.001 p=.973 |  |
| **2E, F** | *Akt3* KO  **Day1 values used for each phase* | Levene’s p=.525 | Training Latency (s) | F(1,36)=.260 p=.613 | F(1,36)=.858 p=.360 | F(1,36)=.133 p=.717 | WT-M=10  KO-M=11  WT-F=10  KO-F=9 |
|  |  | Levene’s p=.071 | Reversal Latency (s) | F(1,36)=.391 p=.536 | F(1,36)=.975 p=.330 | F(1,36)=1.683 p=.203 |  |
|  |  | Levene’s p=.239 | Visible Latency (s) | F(1,36)=.2.318 p=.137 | F(1,36)=.156 p=.696 | F(1,36)=.237 p=.629 |  |
|  |  | Levene’s p=.924 | Probe Quad Time (%) | F(1,36)=.365 p=.549 | F(1,36)=4.011 p=.053 | F(1,36)=.537 p=.468 |  |
|  |  | Levene’s p=.349 | Platform Crossings (#) | F(1,36)=.001 p=.980 | F(1,36)=4.334 p=.045 | F(1,36)=.111 p=.741 |  |
|  |  | Levene’s p=.272 | Distance Swam (cm) | F(1,36)=2.720 p=.108 | F(1,36)=2.698 p=.109 | F(1,36)=.130 p=.721 |  |
|  |  | Levene’s p=.273 | Velocity (cm/s) | F(1,36)=2.720 p=.108 | F(1,36)= 2.697 p=.109 | F(1,36)=.130 p=.721 |  |

| **Figure** | ***Akt* Isoform** | **Homogeneity of variance test** | **Dependent Variable** | **Genotype** | **Sex** | **Interaction (Sex x Genotype)** | **N/group** |
| --- | --- | --- | --- | --- | --- | --- | --- |
| **3A, B** | *Akt1* KO | Levene’s p=.110 | Training pre-CS freezing (%) | F(1,45)=.493 p=.486 | F(1,45)=.1.399 p=.243 | F(1,45)=.3.213 p=.080 | WT-M=16-17  KO-M=13-15  WT-F=10  KO-F=10 |
|  |  | Levene’s p=.267 | Training freezing (%) | F(1,45)=.243 p=.624 | F(1,45)=.836 p=.365 | F(1,45)=2.333 p=.134 |  |
|  |  | Levene’s p=.060 | Context LTM freezing (%) | F(1,48)=3.628 p=.063 | F(1,48)=1.418 p=.240 | F(1,48)=0.107 p=.746 |  |
|  |  | Levene’s p=.060 | Cued LTM pre-CS freezing (%) | F(1,48)=7.829 p=.007 | F(1,48)=1.495 p=.227 | F(1,48)=.035 p=.853 |  |
|  |  | Levene’s p=.460 | Cued LTM freezing (%) | F(1,48)=.147 p=.703 | F(1,48)=2.368 p=.130 | F(1,48)=.170 p=.682 |  |
| **3C, D** | *Akt2* KO | Levene’s p=.580 | Training pre-CS freezing (%) | F(1,44)=.526 p=.472 | F(1,44)=1.006 p=.321 | F(1,44)=.619 p=.436 | WT-M=14  KO-M=14-16  WT-F=10-11  KO-F=10 |
|  |  | Levene’s p=.021 | Training freezing (s) | F(1,44)=2.161 p=.149 | F(1,44)=.1.589 p=.214 | F(1,44)=.347 p=.559 |  |
|  |  | Levene’s p=.406 | Context LTM freezing (%) | F(1,47)=7.229 p=.010 | F(1,47)=1.902 p=.174 | F(1,47)=.273 p=.604 |  |
|  |  | Levene’s p=.109 | Cued LTM pre-CS freezing (%) | F(1,47)=.948 p=.335 | F(1,47)=4.948 p=.031 | F(1,47)=.579 p=.450 |  |
|  |  | Levene’s p=.267 | Cued LTM freezing (%) | F(1,47)=.072 p=.790 | F(1,47)=.485 p=.490 | F(1,47)=.068 p=.795 |  |
| **3E, F** | *Akt3* KO | Levene’s p<.001 | Training pre-CS freezing (%) | F(1,65)=.047 p=.829 | F(1,65)=.274 p=.603 | F(1,65)=12.074 p=.001 | WT-M=18  KO-M=18-20  WT-F=17  KO-F=16-17 |
|  |  | Levene’s p=.016 | Training freezing (%) | F(1,68)=1.277 p=.263 | F(1,68)=.068 p=.795 | F(1,68)=1.071 p=.304 |  |
|  |  | Levene’s p=.535 | Context LTM freezing (%) | F(1,68)=1.919 p=.171 | F(1,68)=1.580 p=.213 | F(1,68)=.150 p=.700 |  |
|  |  | Levene’s p=.070 | Cued LTM pre-CS freezing (%) | F(1,68)=5.942 p=.017 | F(1,68)=.659 p=.420 | F(1,68)=1.100 p=.298 |  |
|  |  | Levene’s p=.524 | Cued LTM freezing (%) | F(1,68)=.424 p=.517 | F(1,68)=.140 p=.709 | F(1,68)=.001 p=.972 |  |

| **Figure** | ***Akt* Isoform** | **Homogeneity of variance test*** | **Dependent Variable** | **Genotype** | **Sex** | **Interaction (Sex x Genotype)** | **N/group** |
| --- | --- | --- | --- | --- | --- | --- | --- |
| **4A, B** | *Akt1* KO  **Day1 values used* | Levene’s p=.158 | D1 pre-CS freezing (%) | F(1,48)=2.760 p=.103 | F(1,48)=.029 p=.867 | F(1,48)=.101  p=.752 | WT-M=17  KO-M=15  WT-F=10  KO-F=10 |
|  |  | Levene’s p=.074 | D1-D3 freezing (%) | F(1,48)=2.664 p=.109 | F(1,48)=5.416 p=.024 | F(1,48)=6.067  p=.017 |  |
|  |  | Levene’s p=.284 | Ext LTM pre-CS freezing (%) | F(1,48)=.527 p=.472 | F(1,48)=.416 p=.522 | F(1,48)=2.501  p=.120 |  |
|  |  | Levene’s p=.114 | Ext LTM freezing (%) | F(1,48)=1.963 p=.168 | F(1,48)=6.904 p=.012 | F(1,48)=4.937  p=.031 |  |
|  |  | Levene’s p=.054 | Renewal pre-CS freezing (%) | F(1,48)=3.944 p=.053 | F(1,48)=2.221 p=.143 | F(1,48)=1.372  p=.247 |  |
|  |  | Levene’s p=.132 | Renewal freezing (%) | F(1,48)=2.011 p=.163 | F(1,48)=3.957 p=.052 | F(1,48)=6.256  p=.016 |  |
| **4C, D** | *Akt2* KO  **Day1 values used* | Levene’s p=.499 | D1 pre-CS freezing (%) | F(1,44)=.320 p=.672 | F(1,44)=.095 p=.809 | F(1,44)=1.344  p=.253 | WT-M=13-14  KO-M=15-16  WT-F=9  KO-F=9 |
|  |  | Levene’s p=.091 | D1-D3 freezing (%) | F(1,42)=.199 p=.658 | F(1,42)=.645 p=.426 | F(1,42)=1.038  p=.314 |  |
|  |  | Levene’s p=.303 | Ext LTM pre-CS freezing (%) | F(1,44)=.001 p=.983 | F(1,44)=.491 p=.611 | F(1,44)=2.795  p=.102 |  |
|  |  | Levene’s p=.402 | Ext LTM freezing (%) | F(1,44)=.134 p=.716 | F(1,44)=8.328 p=.006 | F(1,44)=.000  p=.989 |  |
|  |  | Levene’s p=.019 | Renewal pre-CS freezing (%) | F(1,42) =.404 p=.640 | F(1,42)=4.565 p=.279 | F(1,42)=.493  p=.487 |  |
|  |  | Levene’s p=.414 | Renewal freezing (%) | F(1,42)=3.818 p=.057 | F(1,42)=5.102 p=.029 | F(1,42)=1.635  p=.208 |  |
| **4E, F** | *Akt3* KO  **Day1 values used* | Levene’s p=.001 | D1 pre-CS freezing (%) | F(1,65)=4.460 p=.039 | F(1,65)=1.818 p=.182 | F(1,65)=1.497  p=.226 | WT-M=18  KO-M=20  WT-F=15-17  KO-F=15-16 |
|  |  | Levene’s p=.476 | D1-D3 freezing (%) | F(1,62)=1.841 p=.180 | F(1,62)=.041 p=.840 | F(1,62)=.016  p=.899 |  |
|  |  | Levene’s p=.048 | Ext LTM pre-CS freezing (%) | F(1,65)=.628 p=.431 | F(1,65)=3.453 p=.068 | F(1,65)=1.116  p=.295 |  |
|  |  | Levene’s p=.138 | Ext LTM freezing (%) | F(1,65)=.664 p=.418 | F(1,65)=0.040 p=.843 | F(1,65)=.105  p=.748 |  |
|  |  | Levene’s p=.069 | Renewal pre-CS freezing (%) | F(1,66)=3.393 p=.070 | F(1,66)=.634 p=.429 | F(1,66)=.023  p=.879 |  |
|  |  | Levene’s p=.119 | Renewal freezing (%) | F(1,66)=.078 p=.781 | F(1,66)=.914 p=.343 | F(1,66)=.250  p=.619 |  |

| **Figure** | ***Akt* Isoform** | **Homogeneity of variance test** | **Dependent Variable** | **Genotype** | **Sex (male only)** | **Post hoc testing (Tukey)** | **N/group** |
| --- | --- | --- | --- | --- | --- | --- | --- |
| **5D** | *Akt1* KO *+ AAV-AKT1* | Levene’s p=.389 | Training pre-CS freezing (%) | F(2,34)=.209 p=.812 | N/A | All comparisons p>.05 | WT-Sham=15  KO-Sham=11  KO-*AKT1*=11 |
|  |  | Levene’s p=.571 | Training freezing (%) | F(2,34)=.058 p=.944 | N/A | All comparisons p>.05 |  |
|  |  | Levene’s p=.421 | Context LTM freezing (%) | F(2,34)=.023 p=.977 | N/A | All comparisons p>.05 |  |
|  |  | Levene’s p=.096 | Cued LTM pre-CS freezing (%) | F(2,34)=2.033 p=.146 | N/A | All comparisons p>.05 |  |
|  |  | Levene’s p=.996 | Cued LTM freezing (%) | F(2,34)=1.150 p=.329 | N/A | All comparisons p>.05 |  |
| **5E** | *Akt1* KO *+ AAV-AKT1* | Levene’s p=.299 | D1 pre-CS freezing (%) | F(2,34)=.463 p=.633 | N/A | All comparisons p>.05 | WT-Sham=15  KO-Sham=11  KO-*AKT1*=11 |
|  |  | Levene’s p=.119 | D1-D3 freezing (%) | F(2,34)=5.400 p=.009 | N/A | WT vs KO p=.009  WT vs KO-*AKT1* p=.875  KO vs KO-*AKT1* p=.047 |  |
|  |  | Levene’s p=.165 | Ext LTM pre-CS freezing (%) | F(2,34)=.994 p=.381 | N/A | All comparisons p>.05 |  |
|  |  | Levene’s p=.096 | Ext LTM freezing (%) | F(2,34)=4.388 p=.020 | N/A | WT vs KO p=.035  WT vs KO-*AKT1* p=.985  KO vs KO-*AKT1* p=.037 |  |
|  |  | Levene’s p=.790 | Renewal pre-CS freezing (%) | F(2,29)=.117 p=.890 | N/A | All comparisons p>.05 | WT-Sham=13  KO-Sham=11  KO-*AKT1*=8 |
|  |  | Levene’s p=.243 | Renewal freezing (%) | F(2,29)=1.018 p=.374 | N/A | All comparisons p>.05 |  |

| **Figure** | ***Akt* Isoform** | **Homogeneity of variance test*** | **Dependent Variable** | **Genotype** | **Sex (male only)** | **Interaction (Sex x Genotype)** | **N/group** |
| --- | --- | --- | --- | --- | --- | --- | --- |
| **6A** | *Akt1* cKO | Levene’s p=.205 | Center time (%) | F(1,32)=.024 p=.877 | N/A | N/A | WT=14  cKO=20 |
|  |  | Levene’s p=.074 | Distance moved (cm) | F(1,32)=.464 p=.501 |  |  |  |
| **6B** | *Akt1* cKO | Levene’s p=.565 | Open Arm time (%) | F(1,32)=.041 p=.840 |  |  |  |
|  |  | Levene’s p=.062 | Distance moved (cm) | F(1,32)=.195 p=.662 |  |  |  |
| **6C** | *Akt1* cKO  **Day1 values used for each phase* | Levene’s p=.167 | Training Latency (s) | F(1,14)=.111 p=.744 |  |  | WT=8  cKO=8 |
|  |  | Levene’s p=.664 | Reversal Latency (s) | F(1,14)=.160 p=.695 |  |  |  |
|  |  | Levene’s p=.514 | Visible Latency (s) | F(1,14)=1.001 p=.334 |  |  |  |
|  |  | Levene’s p=.499 | Probe Quad Time (s) | F(1,14)=.008 p=.928 |  |  |  |
|  |  | Levene’s p=1.00 | Platform Crossings (#) | F(1,14)=.836 p=.376 |  |  |  |
|  |  | Levene’s p=.848 | Distance Swam (cm) | F(1,14)=.063 p=.806 |  |  |  |
|  |  | Levene’s p=.846 | Velocity (cm/s) | F(1,14)=.170 p=.686 |  |  |  |
| **6D** | *Akt1* cKO | Levene’s p=.066 | Training pre-CS freezing (%) | F(1,22)=.729 p=.402 |  |  | WT=11  cKO=13 |
|  |  | Levene’s p=.187 | Training freezing (%) | F(1,22)=.035 p=.853 |  |  |  |
|  |  | Levene’s p=.918 | Context LTM freezing (%) | F(1,22)=6.429 p=.019 |  |  |  |
|  |  | Levene’s p=.104 | Cued LTM pre-CS freezing (%) | F(1,22)=1.771 p=.197 |  |  |  |
|  |  | Levene’s p=.098 | Cued LTM freezing (%) | F(1,22)=.678 p=.419 |  |  |  |
| **6E** | *Akt1 c*KO | Levene’s p=.220 | D1 pre-CS freezing (%) | F(1,22)=1.220 p=.281 |  |  | WT=11  cKO=13 |
|  |  | Levene’s p=.318 | D1-D3 freezing (%) | F(1,22)=1.009 p=.326 |  |  |  |
|  |  | Levene’s p=.808 | Ext LTM pre-CS freezing (%) | F(1,22)=.028 p=.868 |  |  |  |
|  |  | Levene’s p=.675 | Ext LTM freezing (%) | F(1,22)=.917 p=.349 |  |  |  |
|  |  | Levene’s p=.130 | Renewal pre-CS freezing (%) | F(1,22)=2.395 p=.136 |  |  |  |
|  |  | Levene’s p=.360 | Renewal freezing (%) | F(1,22)=3.106 p=.092 |  |  |  |
| **supplement 1** | *Akt1* cKO | Levene’s p=.467 | Amygdala AKT1 levels | F(1,10)=36.467 p<.001 |  |  | WT=6  cKO=6 |
|  |  | Levene’s p=.765 | Amygdala AKT3 levels | F(1,10)=.156 p=.701 |  |  |  |
|  |  | Levene’s p=.006 | PFC AKT1 levels | F(1,10)=44.958 p<.001 |  |  |  |
|  |  | Levene’s p=.651 | PFC AKT3 levels | F(1,10)=.253 p=.626 |  |  |  |

| **Figure** | ***Akt* Isoform** | **Homogeneity of variance test*** | **Dependent Variable** | **Genotype** | **Sex** | **Interaction (Sex x Genotype)** | **N/group** |
| --- | --- | --- | --- | --- | --- | --- | --- |
| **7A** | *Akt1* cKO *Akt3* KO | Levene’s p=.583 | Center time (s) | F(1,55)=.772 p=.383 | F(1,55)=.336 p=.547 | F(1,55)=1.613 p=.209 | WT-M=15  cKO-M=13  WT-F=16  cKO-F=15 |
|  |  | Levene’s p=.206 | Distance moved (cm) | F(1,55)=2.139 p=.149 | F(1,55)=.119 p=.732 | F(1,55)=1.763 p=.190 |  |
| **7B** | *Akt1* cKO *Akt3* KO | Levene’s p=.856 | Open Arm time (%) | F(1,55)=.013 p=.908 | F(1,55)=.464 p=.499 | F(1,55)=.613 p=.437 |  |
|  |  | Levene’s p=.927 | Distance moved (cm) | F(1,55)=.581 p=.449 | F(1,55)=.7.246 p=.009 | F(1,55)=.1.952 p=.168 |  |
| **7C, D** | *Akt1* cKO *Akt3* KO  **Day1 values used for each phase* | Levene’s p=.173 | Training Latency (s) | F(1,42)=1.489 p=.229 | F(1,42)=.303 p=.585 | F(1,42)=.178 p=.675 | WT-M=13  cKO-M=9  WT-F=12  cKO-F=12 |
|  |  | Levene’s p=.890 | Reversal Latency (s) | F(1,42)=.407 p=.527 | F(1,42)=.117 p=.734 | F(1,42)=.1.132 p=.293 |  |
|  |  | Levene’s p=.351 | Visible Latency (s) | F(1,42)=.472 p=.496 | F(1,42)=.891 p=.351 | F(1,42)=.289 p=.594 |  |
|  |  | Levene’s p=.915 | Probe Quad Time (s) | F(1,42)=14.393 p<.001 | F(1,42)=.008 p=.931 | F(1,42)=.244 p=.624 |  |
|  |  | Levene’s p=.373 | Platform Crossings (#) | F(1,42)=15.799 p<.001 | F(1,42)=.030 p=.863 | F(1,42)=.463 p=.500 |  |
|  |  | Levene’s p=.730 | Distance Swam (cm) | F(1,42)=.013 p=.911 | F(1,42)=.025 p=.875 | F(1,42)=.719 p=.401 |  |
|  |  | Levene’s p=.797 | Velocity (cm/s) | F(1,42)=.064 p=.802 | F(1,42)=.132 p=.719 | F(1,42)=.607 p=.440 |  |
| **7E, F** | *Akt1* cKO *Akt3* KO | Levene’s p=.003 | Training pre-CS freezing (%) | F(1,43)=4.133 p=.048 | F(1,43)=.003 p=.957 | F(1,43)=.275 p=.602 | WT-M=11  cKO-M=11  WT-F=14  cKO-F=10-11 |
|  |  | Levene’s p=.667 | Training freezing (%) | F(1,43)=.039 p=.844 | F(1,43)=.002 p=.964 | F(1,43)=.121 p=.730 |  |
|  |  | Levene’s p=.731 | Context LTM freezing (%) | F(1,43)=9.660 p=.003 | F(1,43)=.086 p=.770 | F(1,43)=.067 p=.796 |  |
|  |  | Levene’s p=.617 | Cued LTM pre-CS freezing (%) | F(1,42)=.001 p=.979 | F(1,42)=.311 p=.580 | F(1,42)=.590 p=.447 |  |
|  |  | Levene’s p=.992 | Cued LTM freezing (%) | F(1,42)=9.014 p=.004 | F(1,42)=.286 p=.596 | F(1,42)=.002 p=.963 |  |

| For Figures 8 and 9, due to loading number limitations, western blots were run with either all male or all female samples. This allowed for direct comparison of signals between genotype within one sex. Comparing male and female sample signal intensities across independent blots is not possible because of inherent intensity differences between blots. To do so may give the appearance of sex or genotype differences that may in fact be technical artifacts. |
| --- |
